# On the development of B-Raf inhibitors acting through innovative mechanisms

**DOI:** 10.12688/f1000research.108761.1

**Published:** 2022-02-25

**Authors:** Luca Pinzi

**Affiliations:** 1 Department of Life Sciences, University of Modena and Reggio Emilia, Via Giuseppe Campi 103, 41125, Modena, Italy

**Keywords:** B-Raf, allosteric inhibitors, polypharmacology, drug repurposing, PROTACs, drug discovery and development, small molecule inhibitors.

## Abstract

B-Raf is a protein kinase participating to the regulation of many biological processes in cells. Recent studies have demonstrated that this protein is frequently overactivated in human cancers, especially when it bears activating mutations. In recent years, few ATP-competitive inhibitors of B-Raf have been marketed for the treatment of melanoma and are currently under clinical evaluation on a variety of other types of cancer. Although the introduction of drugs targeting B-Raf has provided significant advances in cancer treatment, responses to such ATP-competitive inhibitors remain limited, mainly due to selectivity issues, side effects, narrow therapeutic windows, and the insurgence of drug resistance.

Impressive research efforts have been made so far towards the identification of novel ATP-competitive modulators with improved efficacy against cancers driven by mutant Raf monomers and dimers, some of them showing good premises. However, several limitations could still be envisioned for these compounds, according to recent literature data. Besides, increased attentions have recently arisen around approaches based on the design of allosteric modulators, polypharmacology, PROTACs and drug repurposing for the targeting of B-Raf proteins. The design of compounds acting through such innovative mechanisms is rather challenging. However, novel valuable therapeutic opportunities can be envisioned on these drugs, as they act through innovative mechanisms in which limitations typically observed for approved ATP-competitive B-Raf inhibitors are less prone to emerge. In this article, the most recent approaches adopted for the design of non-ATP competitive inhibitors targeting B-Raf are described, discussing also on the possibilities, ligands acting through such innovative mechanisms could provide for the obtainment of more effective therapies.

The Serine/Threonine protein kinase B-Raf is one among the most widely studied targets for cancer treatment. ^1^^,^^2^ Under physiological conditions, this protein participates as key player in the Ras-Raf-MEK-ERK signaling pathway to the regulation of a number of cellular processes. ^3^^,^^4^ In cancer cells, B-Raf is often upregulated, especially when it bears activating mutations, thus promoting oncogenic cellular processes as uncontrolled proliferation, tumor growth and metastasis. ^5^^–^^9^ Of note, several studies have reported that B-Raf is frequently mutated in human cancers, ^6^^,^^10^^,^^11^ with more than forty oncogenic mutations currently being described for this kinase. ^12^ For these reasons, *wild type* (WT) and mutant B-Raf proteins have gained remarkable relevance for the development of anticancer drugs over the last years. Several drugs selectively targeting B-Raf proteins have also been approved for the treatment of metastatic melanoma, ^13^^,^^14^ providing remarkable advantages in therapeutic regimens, and are currently under evaluation against colorectal cancer. ^15^ However, the therapeutic use of the majority of these drugs is still hampered by drug resistance and side effects issues, resulting in responses that often remain temporary and rarely complete, the median time progression, *e.g.*, for Vemurafenib against melanoma being six to seven months. ^16^ In particular, recent findings have demonstrated that drug resistance often characterizing approved B-Raf inhibitors is mainly driven by feedback deregulation and overexpression of several other kinases. ^17^^–^^20^ Nevertheless, clinical evidences have demonstrated that the use of selected B-Raf inhibitors in therapeutic regiments can also result in Raf paradoxical activation, which promotes cellular hyperproliferation of certain secondary skin lesions. ^21^^–^^26^ In particular, it has been reported that when a B-Raf monomer is bound to specific inhibitors ( *e.g.*, Dabrafenib and Vemurafenib), it can dimerize promoting the aberrant activity of a second drug-free protomer, which cannot be targeted due to protein conformational rearrangements. ^21^ This event in turn promotes abnormal proliferation in cells harboring other oncogenic mutations, through the activation of the MEK–ERK pathway. ^27^ In this context, major research efforts have been devoted so far on the development of novel ATP-competitive kinase inhibitors binding to different αC-helix conformations of B-Raf ( *i.e.*, αC-OUT and αC-IN) ( Figure 1). ^13^^,^^21^^,^^22^^,^^28^^,^^29^ However, the obtainment of clinically safe ATP-competitive inhibitors showing high selectivity towards selected kinases is often elusive. With regards to B-Raf, low efficacy deriving by non-optimal therapeutic windows and the establishment of allosteric priming could be observed, for example, for αC-IN binders, albeit they can abrogate aberrant Raf dimerization-derived activities, through the inhibition of both the monomer and dimer of the kinase. ^13^^,^^30^^,^^31^ Examples of recently reported compounds binding to this type of conformation of B-Raf are the *pan*-Raf inhibitors RAF-265, MLN-2480, TAK-632, LY3009120, CCT196969 and CCT241161, ^32^^–^^34^ some of them having been evaluated in clinical trials ( *e.g.*, ClinicalTrials.gov identifiers: NCT02014116, NCT01425008 and NCT00304525), also in combination with MEK blockers ( *e.g.*, ClinicalTrials.gov identifier: NCT01352273). On the contrary, αC-OUT B-Raf inhibitors demonstrated to provide good selectivity profiles and wider therapeutic windows compared to αC-IN binders. ^13^^,^^13^^,^^28^ Examples of such compounds are, among the others, Vemurafenib, Dabrafenib and Encorafenib, which potently inhibit mutant B-Raf ^V600E^ monomers, but resulted to be ineffective on tumor cellular contexts driven by aberrant Raf dimerization. ^13^^,^^28^ Moreover, compounds binding to the αC-OUT conformation of B-Raf have also been reported to promote paradoxical activation. ^13^^,^^35^ Besides, other studies have been focused on the development of compounds as PLX7904 and PLX8394, able to escape paradoxical activation of B-Raf ( *i.e.*, the so-called “paradox breakers”) and to overcome some of known resistance mechanisms associated to previously reported Raf inhibitors, ^36^^,^^37^ the latter ligand currently being evaluated in Phase I/IIa clinical trials ( ClinicalTrials.gov identifier: NCT02428712). The development of paradox breaker compounds is expected to provide significant impact to anticancer therapy, as they allow to efficiently modulate the activity of mutant B-Raf ^V600E^, while circumventing protein dimerization. ^38^ However, such compounds might also result in limited efficacy towards cancers with mutated Ras, according to recent literature data. ^38^ Despite the advantages that can be observed in latest-generation ATP-competitive inhibitors of this kinase, several evidences encouraged a number of research groups to develop B-Raf modulators acting through innovative mechanisms. These include, for example, the modulation of the kinase activity of B-Raf with type III and IV highly selective allosteric binders, as well as through multi-target approaches ( *i.e.*, polypharmacology) ( Figure 1). The design of allosteric kinase inhibitors of B-Raf is particularly challenging, for example, due to missing activity data or the lack of crystallographic structures suitable for the investigations, whose “ *activation loop*”, “ *activation segment*” and “ *αC-helix*” are often not clearly solved. However, recent advancements in crystallographic and *in silico* techniques will certainly help to overcome several of the issues currently encountered in kinase allosteric ligand design. ^39^^,^^40^ The identification of allosteric inhibitors of B-Raf holds great premises in cancer therapy. Indeed, such targeting approaches are expected to help identifying ligands with higher selectivity towards B-Raf with respect to classic ATP-competitive binders, and to help to overcome drug resistance, similarly to as postulated for other kinases. ^41^^–^^45^ Unfortunately, neither type III, nor type IV small molecule, allosteric modulators of B-Raf have been reported so far. However, potent type III inhibitors binding to an allosteric pocket in proximity to the regulatory αC-helix have been reported for several other kinases, such as BCR-ABL, MEK, EGFR and CDK2, ^46^^–^^52^ providing valuable structural clues for the design of innovative B-Raf modulators. These results have also been fueled by the recent crystallographic resolution of type III allosteric inhibitors of mutant EGFR and MEK ( *e.g.*, see references 45, 49, 53). In this regard, our research group has recently reported the design of previously unseen type III allosteric inhibitors of the CDK2 kinase showing anticancer activity. ^46^^,^^54^ Moreover, we have also reported the identification of structurally novel allosteric modulators of WT and double mutant EGFR, exhibiting inhibitory activity towards non-small cell lung cancer (NSCLC). ^47^ More recently, our research group has demonstrated that B-Raf possesses an allosteric pocket similar to that of EGFR ^T790M^, adopting a DFG-IN/αC-OUT conformation potentially druggable by type III modulators. ^55^ Remarkably, the presence of such allosteric pocket in B-Raf has also been indirectly confirmed in a recent study by Cotto-Rios *et al*. ^56^ In particular, in their study the authors firstly identified Ponatinib as an inhibitor of B-Raf within a drug repositioning campaign, this compound presenting a methyl-piperazine moiety that allocated into the allosteric site in proximity to the kinase regulatory αC-helix. Then, medicinal chemistry optimizations were also performed on Ponatinib, leading to the identification of a compound ( *i.e.*, PHI1) that showed selectivity towards Raf dimers in cancer cells. ^56^ Together with previous considerations and literature data, the results of this study suggest that the design of allosteric inhibitors of B-Raf is feasible. Moreover, these results also suggest that the design of such allosteric ligands would also open to novel strategies enabling the full arrest of the B-Raf kinase activity, potentially either *via* single agents or combination therapies. Nevertheless, the results prospected in this study paved the way towards the identification of innovative B-Raf inhibitors among approved drugs ( *i.e.*, drug repurposing) ( Figure 1), ^56^^,^^57^ this approach being already navigated also with natural products and clinically safe candidates, on different medicinal chemistry research areas. ^58^^–^^64^ Similar considerations can also be argued for the design of allosteric ligands binding to the B-Raf dimer interface ( *i.e.*, type IV). The interest on such type IV allosteric ligands for the targeting of both *wild type* and mutant B-Raf has steadily increased over the past few years, with a number of small polypeptides able to disrupt protein dimerization and transactivation being reported. ^43^^,^^44^^,^^65^^,^^66^ In particular, Beneker *et al.* ^43^ were among the first to report the identification of a small set of polypeptides binding to the Raf dimerization interface. The results achieved in their study not only demonstrated that such a type of targeting is a feasible endeavor on B-Raf, but also that type IV allosteric ligands could provide remarkable results when used in combination with known mutant-selective ATP-competitive inhibitors promoting paradoxical activation of the ERK signaling. ^43^ On the same line, Gunderwala *et al*. ^44^ more recently reported the identification of Braftide, a small polypeptide designed through a computational strategy blocking Raf dimerization. Notably, Braftide demonstrated to efficiently inhibit Raf dimerization and to provide degradation of the MAPK complex. Moreover, Braftide has also proved to synergize with Vemurafenib and Dabrafenib, ^44^ further supporting the potential application of type IV allosteric B-Raf inhibitors on therapeutic regiments with approved ATP-competitive drugs. The possibility to identify allosteric ligands of this kinase could also open to novel therapeutic approaches promoting simultaneous blockade of B-Raf at different sites, for example, if used in combination with approved ATP-competitive drugs. Indeed, such a complementary therapeutic approach is being under study against EGFR-mutant lung cancer, ^52^ and it is expected to provide also valuable opportunities for B-Raf targeting. Allosteric inhibitors acting at a site different to those of the type III and type IV ligands described above have already been investigated for other kinases, in some cases with promising results. ^67^ In the specific case of B-Raf, the identification of these types of inhibitors is still at a preliminary stage, albeit remarkable therapeutic opportunities arising on these grounds could be envisioned for the near future.

**Figure 1.**
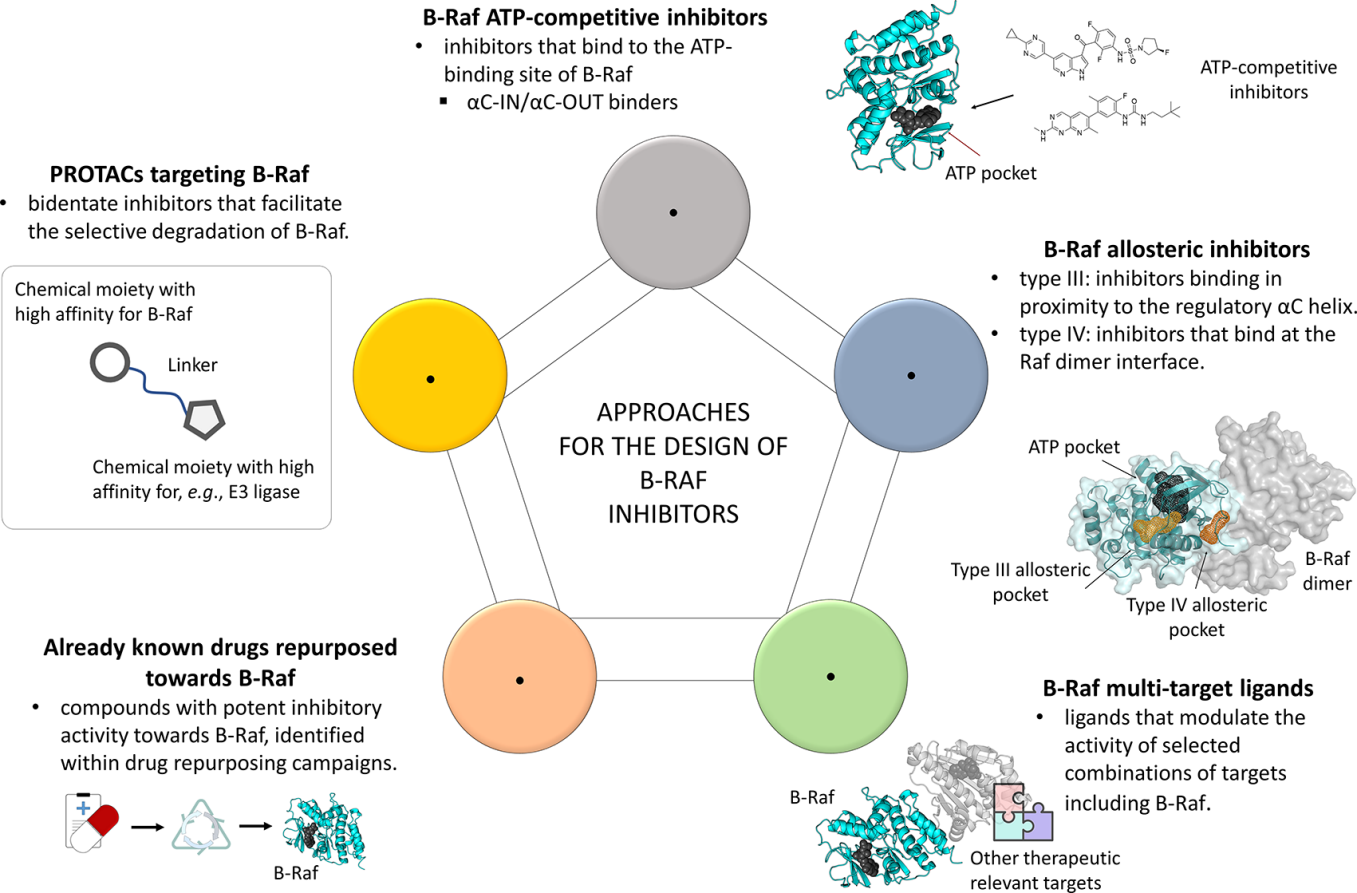
Schematic representation of the approaches currently pursued for the design of B-Raf ligands, discussed in the article.

Novel valuable opportunities could also arise from the identification of compounds exhibiting activity on B-Raf, in selected combinations of targets. The therapeutic advantages deriving by the simultaneous modulation of multiple targets involved in the physiopathology of a disease, either by using a combination of drugs, or with ligands endowed with tailored polypharmacology properties, have already extensively discussed in literature. ^68^^–^^71^ Indeed, the use of approved B-Raf inhibitors is now mainly framed in combined regiments including modulators of other therapeutic relevant targets. For example, the B-Raf ^V600E^ inhibitor Dabrafenib in now mainly used for the treatment of patients with unresectable or metastatic melanoma in combination with Trametinib (a MEK ATP-noncompetitive modulator). ^72^^,^^73^ Similarly, Encorafenib (a B-Raf ^V600E^ inhibitor) is used in combination with Binimetinib for the treatment of the same diseases, since their approval in 2018. ^72^ The importance of B-Raf as a therapeutic relevant target in polytherapies is also testified by the number of combinations including Vemurafenib, Dabrafenib and Encorafenib, with the MEK inhibitors Trametenib and Binimetinib, and Cetuximab that are currently under clinical evaluation, for example, against colorectal cancer ( *e.g.*, ClinicalTrials.gov identifiers: NCT03727763, NCT03693170 and NCT04673955). Besides, several clinical studies have also been reported on the investigation of mutant selective B-Raf inhibitors, with modulators of non-kinase proteins, one among the most studied being Hsp90 ( *e.g.*, ClinicalTrials.gov identifiers: NCT01657591 and NCT02721459). ^68^^–^^70^ The selection of the most suitable targets for combination therapy approaches is generally driven by their involvement in relevant oncogenic processes. For example, several mechanisms by which tumor cells can exert drug resistance to B-Raf inhibitors derive by deregulation or overexpression of other oncoproteins, ^18^^–^^20^^,^^74^^,^^75^ many of them being “ *clients*” of Hsp90. ^76^^,^^77^ Consequently, the simultaneous targeting of Hsp90 and B-Raf have represented an attractive strategy to overcome drug resistance to B-Raf inhibitors so far. Indeed, several biological studies and clinical evidence demonstrated that the inhibition of Hsp90 helps to overcome resistance to known blockers of B-Raf, and that their combined inhibition provides synergistic effects in different cancer-related contexts. ^78^^–^^80^ In line with the polypharmacology concept, further advantages can also be envisioned for cancer treatment on the design of ligands endowed with multi-target activity ( Figure 1).

In the case of B-Raf, several studies describing the design of multi-target ligands have been reported so far. ^81^^–^^86^ In particular, Anighoro *et al*. ^81^ reported in 2017 the identification of the first two compounds endowed with activity towards B-Raf and Hsp90, demonstrating that these targets share overlapping chemical spaces. The compounds reported in this study were identified by means of an integrated *in silico* strategy and represent valuable starting points for the development of innovative B-Raf/Hsp90 dual inhibitors, especially considering that they showed balanced multi-target activity and low molecular weight. Of note, Hsp90 and B-Raf belong to different protein families and present distinct binding site architectures, which makes the design of dual ligands of these proteins a difficult task. The interest around Hsp90 and B-Raf as partners in polypharmacology strategies has also been further explored more recently within an effort to identify ligands endowed with Hsp90/PDHK1/B-Raf multi-target activity. ^82^ The identification of compounds with such a tailored polypharmacology profile would enable the modulation of multiple pathways important to survival and proliferation of tumor cells, thus resulting in more effective anticancer therapies. However, the obtainment of Hsp90/PDHK1/B-Raf multi-target inhibitors is very challenging, as several, often conflicting, structural requirements should be taken into account in the ligand design process.

Besides, B-Raf has also been framed into multi-target ligand design projects including other kinases, such as VEGFR-2, p38α and EGFR. ^83^^–^^86^ In particular, several studies reported the identification of dual inhibitors of the B-Raf and VEGFR-2 kinases. ^85^^,^^87^^,^^88^ The rationale behind the selection of B-Raf and VEGFR-2 for the development of multi-target inhibitors stands on the fact that these proteins fulfill complementary leading roles on processes related to cancer development and progression. ^89^^,^^90^ For similar reasons, research efforts have also been performed for the design of B-Raf/p38α dual inhibitors. ^86^^,^^91^ Of note, continuous research has also been done for the targeting B-Raf and EGFR, either *via* combination of selective kinase inhibitors, or with polypharmacology ligands. For example, the inhibition of these targets has already been explored on colorectal cancer by means of combination of drugs, as drug resistance that derives by overexpression and activation of EGFR could be overcome through the blockage of B-Raf, according to recent studies. ^20^^,^^92^ The design of B-Raf/EGFR dual inhibitors has also been probed as a strategy to overcome drug resistance observed on melanoma and colorectal cancers to approved B-Raf ^V600E^ drugs, providing promising results. Whereas reservations have been very recently raised on the dual inhibition of these targets as a therapeutic opportunity for NSCLC patients. ^93^ Although particularly challenging, possibilities on the identification of innovative B-Raf/EGFR dual inhibitors could also be envisioned on type III allosteric contexts. Indeed, B-Raf and EGFR present structurally similar type III allosteric pockets, ^55^ which make them ideal candidates for the design of multi-target ligands, for example, by means of computational structure-based approaches as docking. ^94^^,^^95^

In recent years, increased research interests have also arisen around PROTACs ( *i.e.*, proteolysis targeting chimeras) for targeting several therapeutic targets ( Figure 1). ^14^^,^^96^^,^^97^ Such an approach generally employs bidentate molecules bearing to two covalently bounded chemical moieties, one with high affinity towards the target of interest and the other recruiting specific components of the proteasomal degradation system, to promote selective intracellular proteolysis ( *e.g.*, an E3 ligase as VHL). ^98^^,^^99^ At present, PROTACs with high substrate specificity have been reported for targeting different protein kinases, ^14^ including also mutant B-Raf. ^14^^,^^100^^,^^101^ One example of such B-Raf mutant-selective PROTACs comes from a recent study by Alabi *et al*., ^102^ in which the authors designed a compound ( *e.g.*, SJF-0628) showing high selectivity towards degradation of B-Raf ^V600E^. Although being still at their infancy, approaches based on the targeting of B-Raf with PROTACs technology are expected to provided novel valuable opportunities for cancer treatment. Indeed, such approaches allow to also promote complete removal of the protein scaffold other than blocking its catalytic functions, which might represent a valuable advantage over already reported ATP-competitive inhibitors.

Targeting protein kinases, such as B-Raf, has provided several therapeutic advantages in cancer treatment so far, as also testified by the number of approved drugs and clinical candidates modulating the activity of these proteins that are currently under investigation. ^14^ B-Raf has acted as a major player in this context, with some of its ATP-competitive inhibitors ( *e.g.*, Sorafenib, Vemurafenib, Dabrafenib and Encorafenib) being approved for the treatment of patients with unresectable or metastatic melanoma in the last two decades. ^14^ Although B-Raf ^V600E^ inhibitors provided remarkable advantages in anticancer therapeutic regimens, several limitations could still be envisioned for these compounds, the most relevant being the establishment of drug resistance, paradoxical activation mediated by Raf dimerization and transactivation, and low efficacy towards Ras mutated cancers. ^38^ Different strategies based on classic ATP-competitive single-targeting approaches are still being under study to overcome such limitations, some of them showing good premises. ^13^ However, novel, and perhaps more valuable, opportunities can be envisioned on approaches targeting the allosteric sites of B-Raf proteins. Indeed, such approaches have already demonstrated to provide remarkable opportunities on other therapeutic-relevant kinases exhibiting high structural similarity with B-Raf. ^52^ Moreover, the activity of allosteric kinase inhibitors is less prone to be affected by insurgence of drug resistance deriving by site point mutations with respect to ATP-competitive binders, ^41^ this being a significant advantage in anticancer therapy. Importantly, the modulation of B-Raf by means of allosteric ligands would open to complementary approaches including also already reported ATP-competitive inhibitors to promote more efficient arrest the kinase activity, which is expected to result in improved therapeutic outcomes. ^43^^,^^44^ In the near future, increasing research efforts will also be addressed towards the identification of multi-target inhibitors modulating B-Raf activity. Indeed, the importance of polypharmacology approaches for kinase targeting is well established, ^103^ as also testified by the increasing number of dual inhibitors targeting B-Raf reported in the literature over the last years. ^81^^–^^86^ The selection of suitable combinations of targets for the rational design of B-Raf polypharmacology ligands is of primary importance in this context, the identification of those providing the highest therapeutic effectiveness being very often difficult. However, recent innovations on computational approaches are expected to aid on their identification. ^104^ Similar considerations can also be drawn for the identification of inhibitors targeting B-Raf through innovative mechanisms among already approved drugs, as recently observed for Ponatinib. ^56^ Moreover, approaches based on PROTACs technology are also expected to bring significant chemical novelty on future B-Raf inhibitors design, as such compounds exert their activity through molecular mechanisms that are completely different with respect to those of approved drugs and compounds under investigation. The design of either allosteric, polypharmacology or PROTAC ligands targeting B-Raf proteins is challenging, especially with respect of classic kinase APT-competitive binders. However, the recent advancements on understanding cancer cells biology and the improvements on experimental techniques and *in silico* approaches available for the analysis of information reported in public databases, will certainly facilitate the identification of novel B-Raf inhibitors acting through such innovative mechanisms.

## Data availability

No data are associated with this article.

## Author contributions

L.P.: Conceptualization, Writing – Original Draft Preparation, Writing – Review & Editing.
