# Supplementary material for: On the development of B-Raf inhibitors acting through innovative mechanisms
Source: F1000Res. 2022 Apr 26;11:237. Originally published 2022 Feb 25. [Version 2] doi: 10.12688/f1000research.108761.2 (PMC9043679; doi:10.12688/f1000research.108761.2)
Supplement: Peer review history

# Peer review: Reviewer response for version 2

**DOI:** 10.5256/f1000research.132428.r135948

**Published:** 2022-04-26

**Authors:** Andrew Anighoro

**Affiliations:** 1 Relation Therapeutics LTD, London, UK

The author has appropriately and extensively addressed my previous review. I have no further comments.

Is the topic of the opinion article discussed accurately in the context of the current literature?

Yes

Are arguments sufficiently supported by evidence from the published literature?

Yes

Are all factual statements correct and adequately supported by citations?

Yes

Are the conclusions drawn balanced and justified on the basis of the presented arguments?

Partly

Reviewer Expertise:

Drug discovery, computational chemistry.

I confirm that I have read this submission and believe that I have an appropriate level of expertise to confirm that it is of an acceptable scientific standard.

# Peer review: Reviewer response for version 1

**DOI:** 10.5256/f1000research.120182.r125513

**Published:** 2022-03-15

**Authors:** Andrew Anighoro

**Affiliations:** 1 Relation Therapeutics LTD, London, UK

The author gives an overview of B-Raf as a known target for cancer related diseases. Then, provides an account of design strategies that may overcome some limitations of typical ATP competitive drugs.

- The author mostly refers to B-Raf as a target validated by "recent" evidence/studies, while it has been described in the literature as an oncogene at least from the 90s and Vemurafenib began clinical testing in 2008 to be approved in 2011 by FDA followed shortly by other drugs. I don't think this makes the article less interesting, but I feel that B-Raf should be referred sometimes in the text with adjectives such as "well established" rather than "recent".
- Would it be possible to give the reader an idea of how the αC-helix is structurally related to the ATP binding site? Maybe through a sentence in the text and/or marking it in Figure 1?
- When writing “ *activation loop*”, “ *activation segment*” and “ *αC-helix*” are italics and quotes necessary? αC-helix has already been used several times in the text up until then without quotes or italics.
- I think that the sentence " *recent advancements in crystallographic and in silico techniques will certainly help to overcome several of the issues currently encountered in kinase allosteric ligand design*" should be followed by at least mentioning some examples of techniques that the author is certain are going to help.
- Can it be explained more explicitly why Ponatinib/PHI1 do not qualify as allosteric inhibitors?
- Would it be possible to include a comment on the challenge of rationally designing kinase multitarget inhibitors given the selectivity issues mentioned by the author? 
- The first time that the term PROTAC is mentioned, it should be by the full name followed by the abbreviation, proteolysis targeting chimera (PROTAC).
- "( *e.g.*, an E3 ligase as VHL)" Probably, CRBN should be mentioned as well. Both should be mentioned by their full name first.
- Probably, "e.g." in Figure 1 when addressing E3 ligases can be removed as it makes it look like E3 ligases can be replaced by something else in the context of PROTACs. I am not aware if that's actually possible, but if that's the case, it should be mentioned in the text.  
- In the abstract and in the main text the word premises is used a few times instead of promise, I believe.

Is the topic of the opinion article discussed accurately in the context of the current literature?

Yes

Are arguments sufficiently supported by evidence from the published literature?

Yes

Are all factual statements correct and adequately supported by citations?

Yes

Are the conclusions drawn balanced and justified on the basis of the presented arguments?

Partly

Reviewer Expertise:

Drug discovery, computational chemistry.

I confirm that I have read this submission and believe that I have an appropriate level of expertise to confirm that it is of an acceptable scientific standard, however I have significant reservations, as outlined above.

# Response

**Published:** 2022-04-16

**Authors:** Luca Pinzi

**Affiliations:** University of Modena and Reggio Emilia, Italy

I would like to thank the Reviewer for the comments and the precious suggestions. The manuscript was revised to better contextualize the development of innovative B-Raf targeting approaches, with respect to established efforts (e.g., ATP-competitive inhibitors development). Accordingly, the descriptions referring to literature data were also revised under a more contemporary perspective.

 A brief description of the main elements characterizing the ATP-binding site of the kinases was reported in the revised version of the manuscript. This helped to delineate how the regulatory αC-helix is related to the other structural regions in the kinases ATP binding site. Besides, Figure 1 was also revised to better highlight the position of the αC-helix, with respect to the hinge region in the B-Raf active site to which ATP-competitive inhibitors bind to, and to improve the image contents.   

 The text was also revised to provide further insights on some of the experimental techniques and *in silico* approaches that are (and are also expected to be even more in the future) of help for kinase allosteric drug design efforts. Moreover, additional references to relevant literature data were included in the revised version of the manuscript to better contextualize the application of some of these techniques, on both B-Raf related research and a more general perspective.

 An explanation of how Ponatinib and PHI1 can be classified according to their experimentally derived binding mode is now provided in the text, as suggested by the Reviewer. As revised, the text is expected to help better contextualize the example reported on Ponatinib/PHI1, with respect to the type III B-Raf allosteric pocket mentioned within this article.

 The main challenges that might be faced when rationally designing multi-kinase inhibitors are now discussed in the text. Moreover, the main E3 ligases that have been exploited so far for the design of PROTACs are now reported within the text.

 The manuscript was carefully revised, to remove typos and to make it clearer to readers. Moreover, the full names of the reported abbreviations were added within the text where needed.

# Peer review: Reviewer response for version 1

**DOI:** 10.5256/f1000research.120182.r125510

**Published:** 2022-03-04

**Authors:** Federico Falchi

**Affiliations:** 1 Laboratory of Computational Medicinal Chemistry, Department of Pharmacy and Biotechnology, University of Bologna, Bologna, Italy

In this nice perspective, Pinzi summarizes the current state-of-the-art in the development of B-Raf inhibitors acting through innovative mechanisms. After a brief introduction about the traditional inhibitors (Atp-competitive) with their big limitations, Pinzi describes the most challenging approaches such as the design of allosteric modulators, the polypharmacology, and the PROTACs design with an eye to the computational strategies.

 The manuscript is well understandable, offering both a biological and medicinal chemistry point of view. The references are adequate.

 I do believe that work can be accepted certainly for indexing.

 I have only a very few minor remarks:

1. The sentence below is too long:
  Besides, other studies have been focused on the development of compounds as PLX7904 and PLX8394, able to escape paradoxical activation of B-Raf (i.e., the so-called “paradox breakers”) and to overcome some of known resistance mechanisms associated to previously reported Raf inhibitors,36,37 the latter ligand currently being evaluated in Phase I/IIa clinical trials (ClinicalTrials.gov identifier: NCT02428712).
  Please change for example as:
  Besides, other studies have been focused on the development of compounds such as PLX7904 and PLX8394, able to escape paradoxical activation of B-Raf (i.e., the so-called “paradox breakers”) and to overcome some of the known resistance mechanisms associated with previously reported Raf inhibitors.
  PLX8394 is currently being evaluated in Phase I/IIa clinical trials (ClinicalTrials.gov identifier: NCT02428712).
2. The sentence below is too long:
  Nevertheless, the results prospected in this study paved the way towards the identification of innovative B-Raf inhibitors among approved drugs (i.e., drug repurposing) (Figure 1), this approach being already navigated also with natural products and clinically safe candidates, on different medicinal chemistry research areas
  Please change for example as:
  Nevertheless, the results prospected in this study paved the way towards the identification of innovative B-Raf inhibitors among approved drugs (i.e., drug repurposing) (Figure 1). This approach is already been conducted also with natural products and clinically safe candidates in different medicinal chemistry research areas.
3. The abbreviation NSCLC is not straightforward, the first time an abbreviation is used it should be explained.
4. In the image:
  - a B in B-RAF is missing 
  - the bullets points are not all the same
  - sometimes a period is used at the end of the sentence, other times not 

Is the topic of the opinion article discussed accurately in the context of the current literature?

Yes

Are arguments sufficiently supported by evidence from the published literature?

Yes

Are all factual statements correct and adequately supported by citations?

Yes

Are the conclusions drawn balanced and justified on the basis of the presented arguments?

Yes

Reviewer Expertise:

Drug design and drug development by means of computational techniques.

I confirm that I have read this submission and believe that I have an appropriate level of expertise to confirm that it is of an acceptable scientific standard.

# Response

**Published:** 2022-04-16

**Authors:** Luca Pinzi

**Affiliations:** University of Modena and Reggio Emilia, Italy

I would like to thank the Reviewer for their precious comments. The manuscript was carefully revised, and long sentences split to make the text clearer to readers, in agreement to the Reviewer suggestions. Moreover, the full name of the reported abbreviations was added in the text. Figure 1 was revised to remove typos as suggested and to improve the image contents.
